# Supplementary material for: The Antioxidant Effects of Trypsin-Hydrolysate Derived from Abalone Viscera and Fishery By-Products, and the Angiotensin-I Converting Enzyme (ACE) Inhibitory Activity of Its Purified Bioactive Peptides
Source: Mar Drugs. 2024 Oct 7;22(10):461. doi: 10.3390/md22100461 (PMC11509546; doi:10.3390/md22100461)
Supplement: Supplementary file 1 [file marinedrugs-22-00461-s001.zip › marinedrugs-3243799-supplementary.pdf]

Supplementary Information

The Antioxidant Effects of Trypsin-Hydrolysate Derived from Abalone Viscera and Fishery By-Products, and the Angioten-sin-I Converting Enzyme (ACE) Inhibitory Activity of Its Purified Bioactive Peptides

Jun-Ho Heo [1], Eun-A Kim [1], Nalae Kang [1], Seong-Yeong Heo [1,2], Ginnae Ahn [3] and Soo-Jin Heo [1,2,*]

[1] Jeju Bio Research Center, Korea Institute of Ocean Science and Technology (KIOST), Jeju 63349, Republic of Korea; unknown0713@kiost.ac.kr (J.-H.H.); euna0718@kiost.ac.kr (E.-A.K.); nalae1207@kiost.ac.kr (N.K.); syheo@kiost.ac.kr (S.-Y.H.)

[2] Department of Marine Biology, University of Science and Technology, Daejeon 34113, Republic of Korea

[3] Department of Food Technology and Nutrition, Chonnam National University, Yeosu 59626, Republic of Korea; gnahn@jnu.ac.kr

* Correspondence: sjheo@kiost.ac.kr

Figure S1. Prediction of the binding site of ACE-bioactive peptides complexes. The ACE-bioactive peptide complexes are shown by favorable hydrogen bond interactions at overview and specific point

Figure S2. Sites formed by selecting residues involved in the interaction. The sites were formed using the define site tool of DS that creates a sphere with a minimum radius is 5 Å around the centroid of selected objects with the radius adjusted to include them. The spheres centered around residues in the active sites pocket: Red-S1, Green-S1', and Blue-S2 (A). The sphere formed around residues' interaction in binding to the zinc ion (B).

Figure S3. Prediction of the binding site of ACE-lisinopril and captopril complexes. The complexes are shown by favorable hydrogen bond interactions at overview and interaction represented as stick models of residues with amino acid names and 2D diagrams of the binding complex.

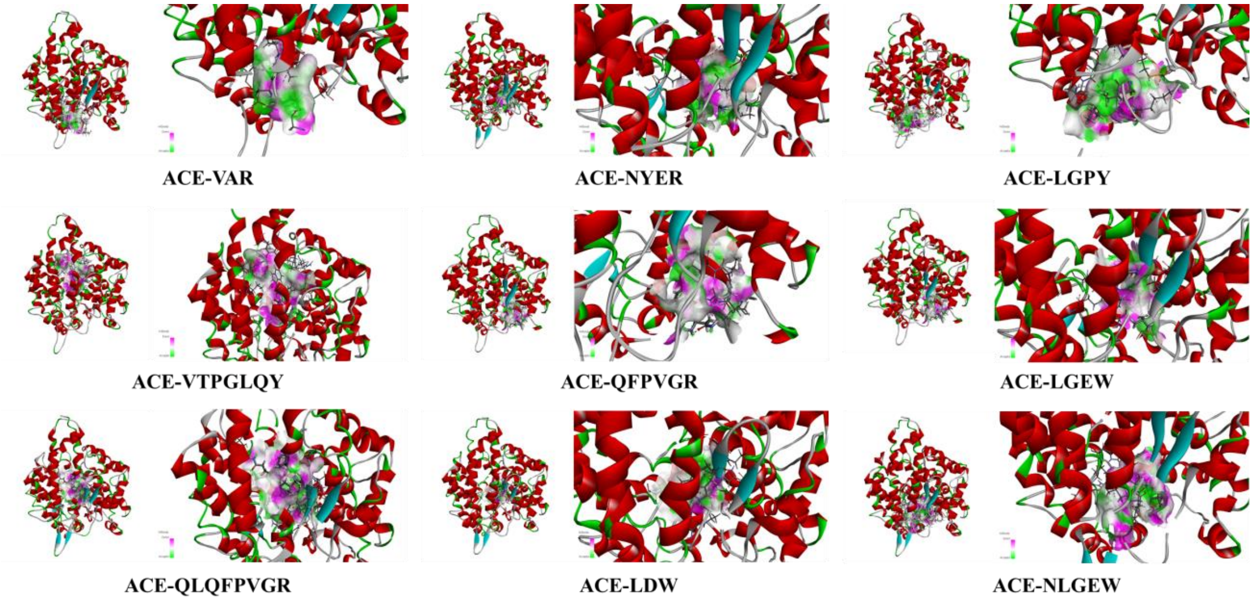

Figure S1. Prediction of the binding site of ACE-bioactive peptides complexes. The ACE-bioactive peptide complexes are shown by favorable hydrogen bond interactions at overview and specific point

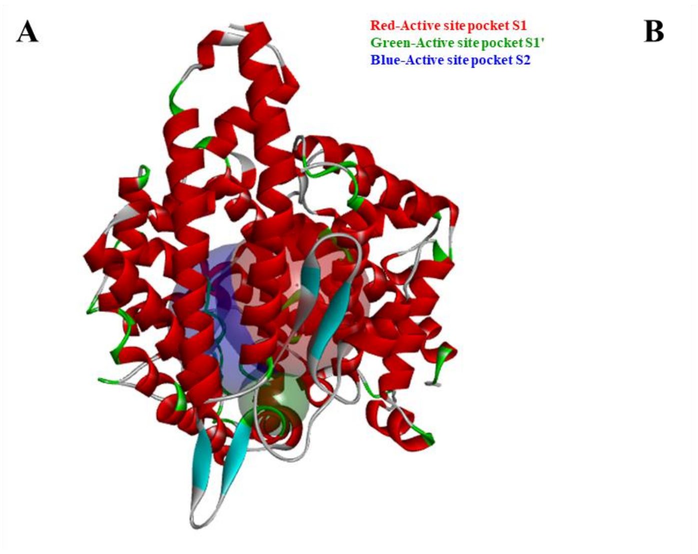

Figure S2. Sites formed by selecting residues involved in the interaction. The sites were formed using the define site tool of DS that creates a sphere with a minimum radius is 5 Å around the centroid of selected objects with the radius adjusted to include them. The spheres centered around residues in the active sites pocket: Red-S1, Green-S1', and Blue-S2 (A). The sphere formed around residues' interaction in binding to the zinc ion (B).

ACE-lisinopril

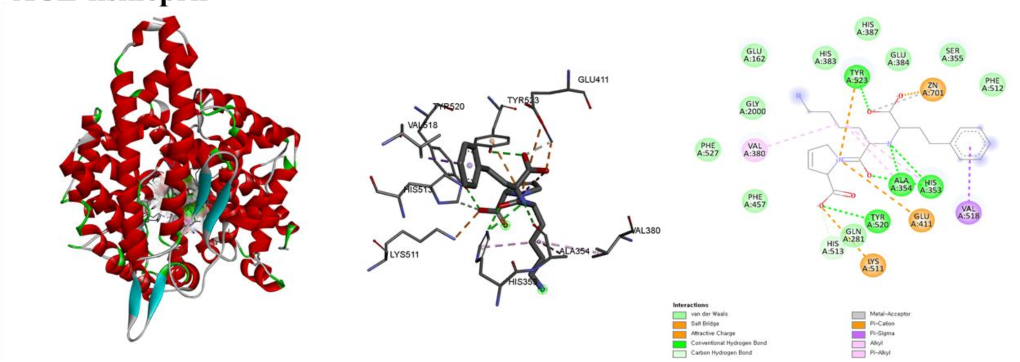

ACE-captopril

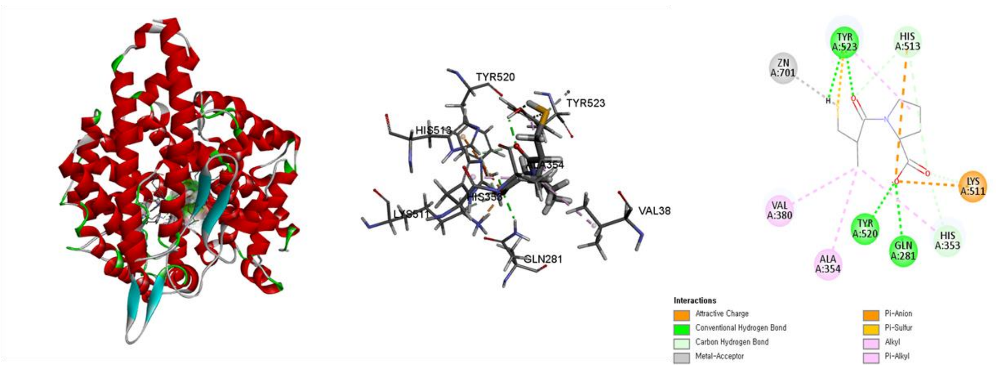

Figure S3. Prediction of the binding site of ACE-lisinopril and captopril complexes. The complexes are shown by favorable hydrogen bond interactions at overview and interaction represented as stick models of residues with amino acid names and 2D diagrams of the binding complex.
